# Supplementary figures and images for: Circulating serum miRNAs predict response to platinum chemotherapy in high‐grade serous ovarian cancer
Source: Cancer Med. 2024 Nov 25;13(22):e70251. doi: 10.1002/cam4.70251 (PMC11588858; doi:10.1002/cam4.70251)

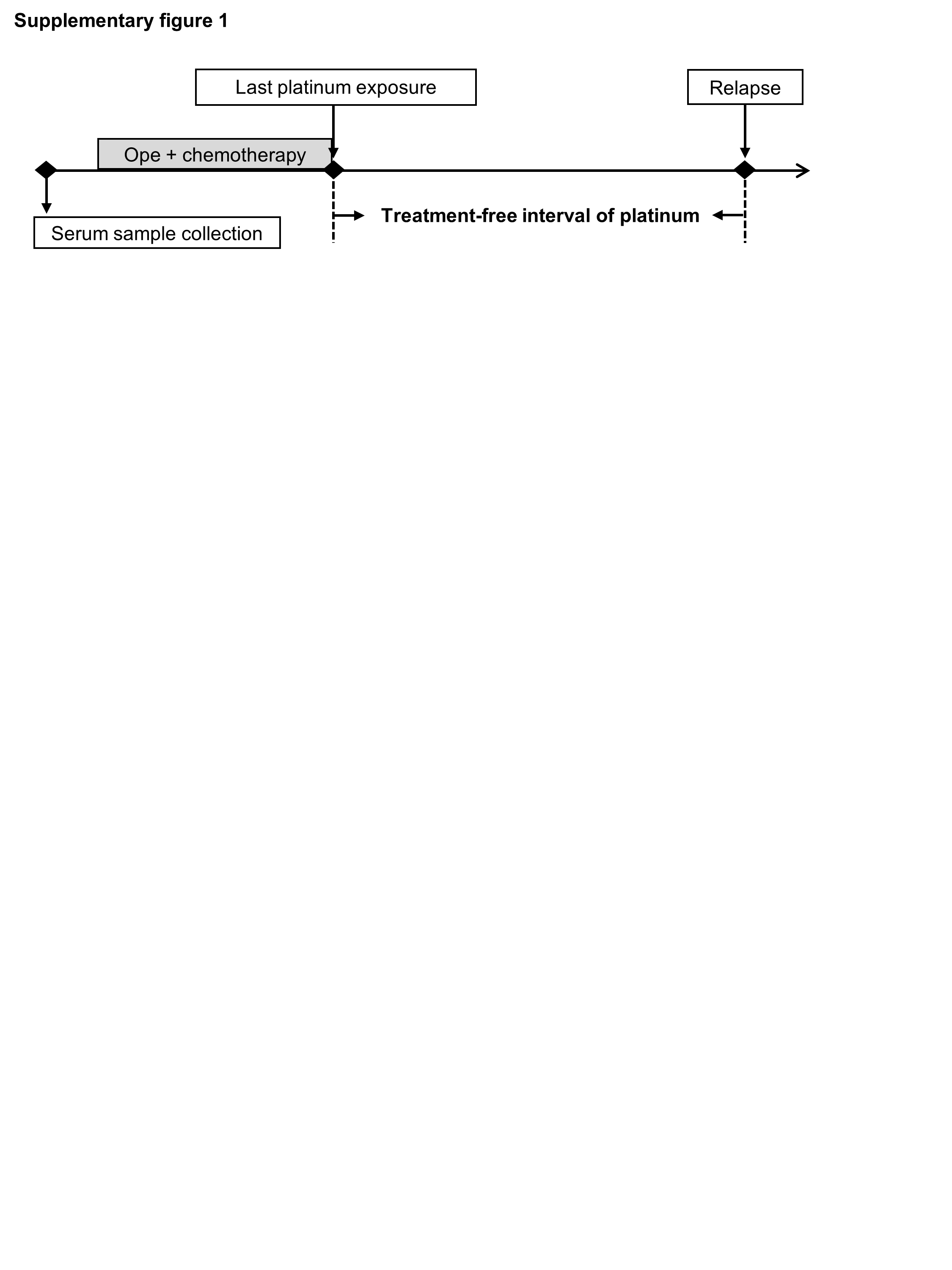

Supplement: Supplementary file 1 — Figure S1. Time course of ovarian cancer treatment and definition of treatment‐free interval of platinum. Abbreviation: Ope, operation. [file CAM4-13-e70251-s001.tif]

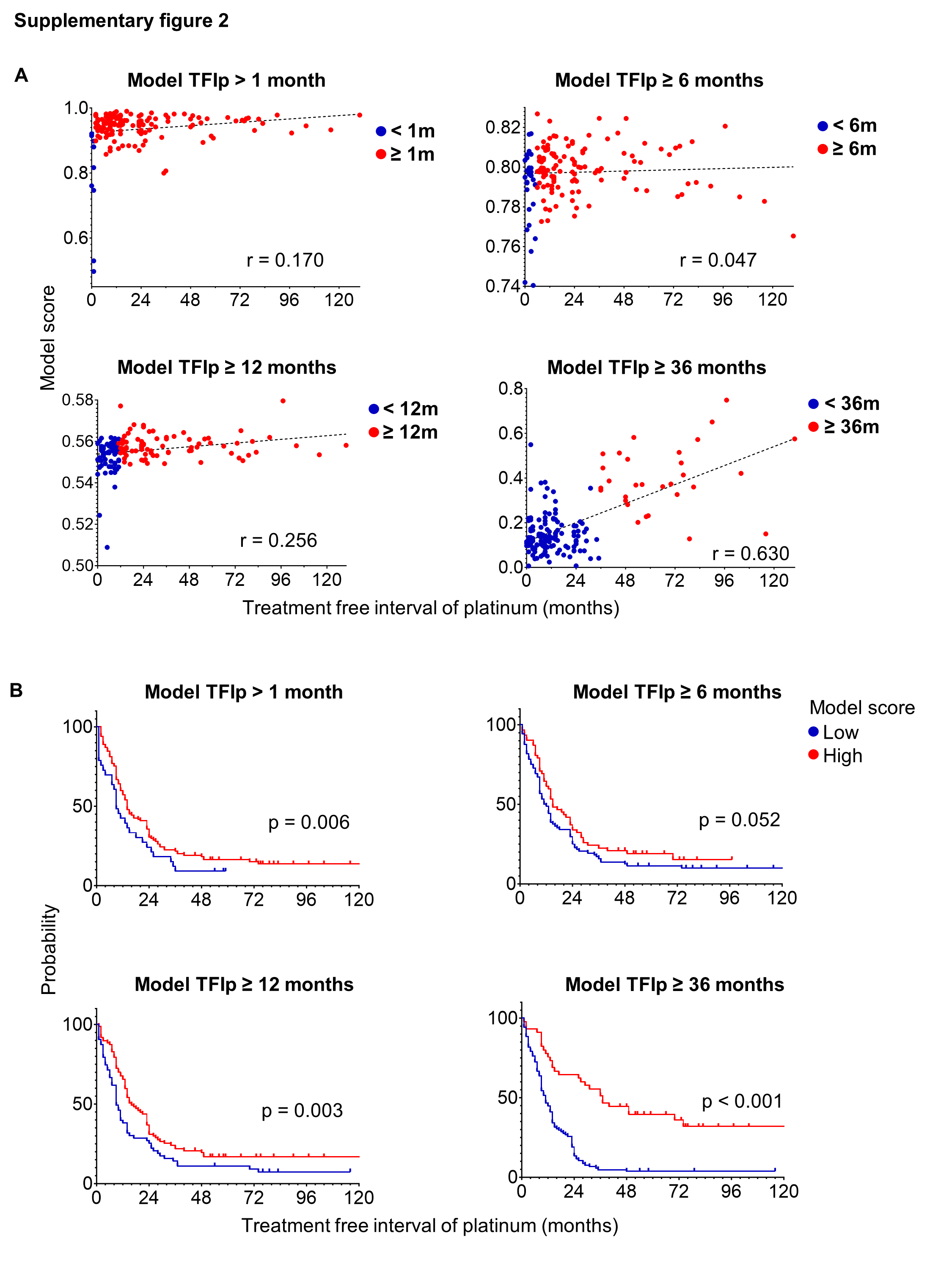

Supplement: Supplementary file 2 — Figure S2. (A). Dot plot for each model. Dotted lines indicate linear regression model. R values were calculated using Pearson’s correlation coefficient. (B). Events over time were evaluated based on predictive models. Kaplan–Meier curves showing patients stratified into high and low groups by each model with cutoffs based on Youden index. p values were calculated using the generalized Wilcoxon test. [file CAM4-13-e70251-s002.tif]

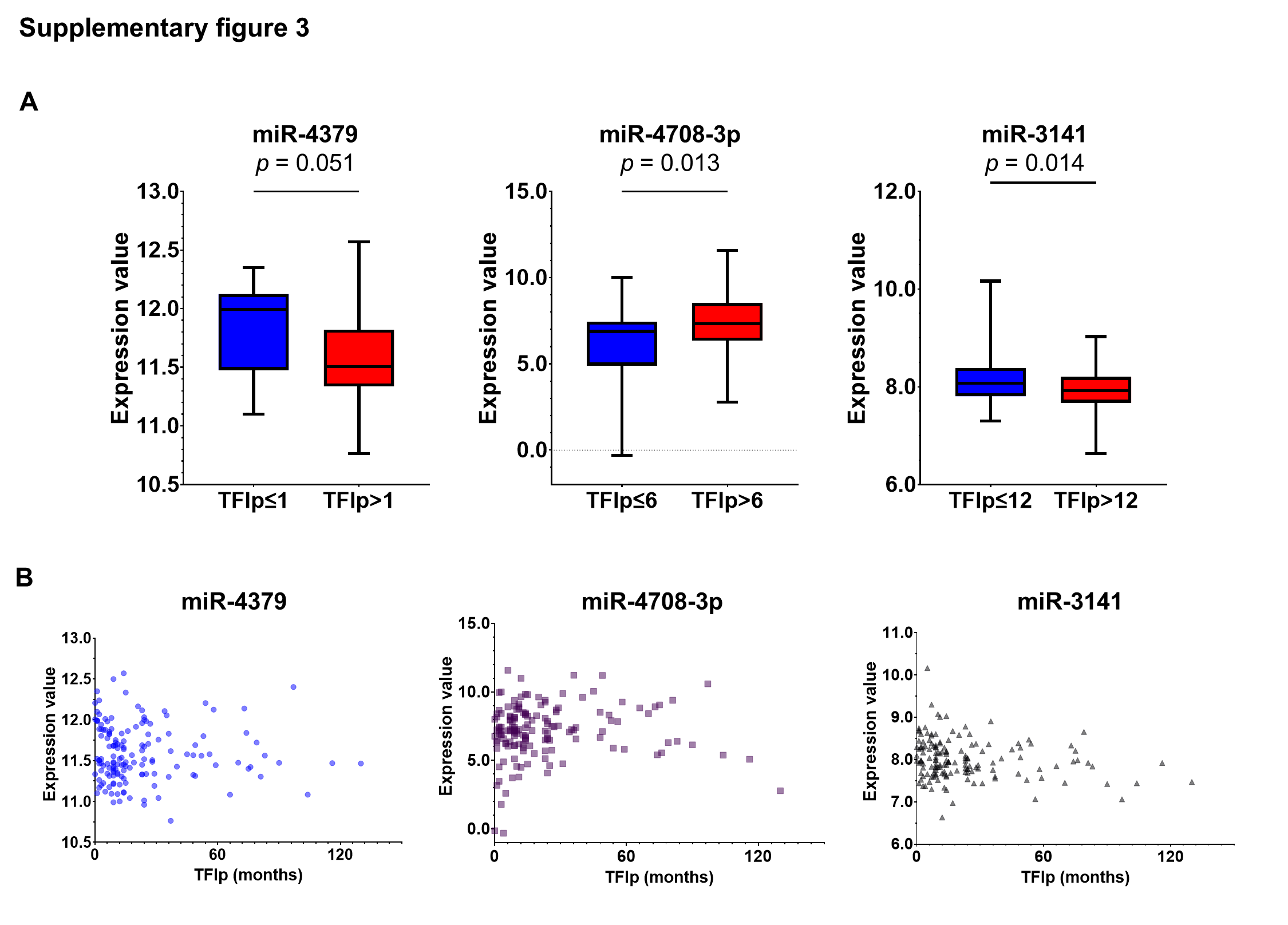

Supplement: Supplementary file 3 — Figure S3. The expression levels of miRNAs. [file CAM4-13-e70251-s003.tif]
