# Supplementary material for: Circulating serum miRNAs predict response to platinum chemotherapy in high‐grade serous ovarian cancer
Source: Cancer Med. 2024 Nov 25;13(22):e70251. doi: 10.1002/cam4.70251 (PMC11588858; doi:10.1002/cam4.70251)
Supplement: Supplementary file 4 — Table S1. [file CAM4-13-e70251-s004.docx]

Supplementary Table1. Detail of predictive model

| Model No. | Predictive model Y |
| --- | --- |
| 1-1 | (-0.08022 × miR-1228-5p) + (-0.16169 × miR-1273g-3p) + (0.23871 × miR-3917) + (-0.0793 × miR-3940-5p) + (-0.72774 × miR-4739) + (0.3835 × miR-498) + (0.01805 × miR-6088) + (0.04429 × miR-6872-3p ) + 10.07757 |
| 1-2 | (-0.305235 × miR-1228-5p) + (-0.2308 × miR-1273g-3p) + (0.271838 × miR-3917) + (-0.13299 × miR-3940-5p) + (0.005181 × miR-4708-3p) + (-0.89869 × miR-4739) + (0.435498 × miR-498) + (0.231217 × miR-6088) + (0.085698 × miR-6872-3p ) + 12.543835 |
| 1-3 (**Model TFIp > 1 month**) | (-0.43074 × miR-1228-5p) + (-0.2713 × miR-1273g-3p) + (0.2881 × miR-3917) + (-0.15449 × miR-3940-5p) + (0.03679 × miR-4708-3p) + (-1.00381 × miR-4739) + (-0.0285 × miR-486-5p) + (0.47157 × miR-498) + (0.35716 × miR-6088) + (0.09762 × miR-6872-3p ) + 13.9039 |
| 1-4 | (-0.58016 × miR-1228-5p) + (-0.307222 × miR-1273g-3p) + (0.330061 × miR-3917) + (-0.171047 × miR-3940-5p) + (0.092346 × miR-4708-3p) + (-1.1506 × miR-4739) + (-0.142806 × miR-486-5p) + (0.526739 × miR-498) + (0.007233 × miR-6087) + (0.525026 × miR-6088) + (0.107553 × miR-6872-3p ) + 15.494438 |
| 1-5 | (-0.646803 × miR-1228-5p) + (-0.313229 × miR-1273g-3p) + (0.030311 × miR-371a-5p) + (0.34458 × miR-3917) + (-0.165331 × miR-3940-5p) + (0.002726 × miR-4449) + (0.110589 × miR-4708-3p) + (-1.211764 × miR-4739) + (-0.178796 × miR-486-5p) + (0.545045 × miR-498) + (0.032721 × miR-6087) + (0.569194 × miR-6088) + (0.11015 × miR-6872-3p ) + 15.78128 |
| 1-6 | (-0.90035 × miR-1228-5p) + (-0.33003 × miR-1273g-3p) + (-0.01715 × miR-17-3p) + (0.13348 × miR-371a-5p) + (0.36831 × miR-3917) + (0.05396 × miR-3928-3p) + (-0.20548 × miR-3940-5p) + (0.15664 × miR-4449) + (0.16038 × miR-4708-3p) + (-1.45235 × miR-4739) + (-0.27004 × miR-486-5p) + (0.57565 × miR-498) + (0.74862 × miR-6088) + (0.11426 × miR-6872-3p ) + 18.19444 |
| 1-7 | (-0.002713 × miR-1181) + (-0.991055 × miR-1228-5p) + (-0.328202 × miR-1273g-3p) + (-0.037427 × miR-17-3p) + (0.169809 × miR-371a-5p) + (0.377633 × miR-3917) + (0.079125 × miR-3928-3p) + (-0.222452 × miR-3940-5p) + (0.196208 × miR-4449) + (0.17659 × miR-4708-3p) + (-1.538496 × miR-4739) + (-0.292685 × miR-486-5p) + (0.58726 × miR-498) + (0.80589 × miR-6088) + (0.112803 × miR-6872-3p ) + 19.075818 |
| 1-8 | (-0.009675 × miR-1181) + (-1.050908 × miR-1228-5p) + (-0.331264 × miR-1273g-3p) + (-0.0542 × miR-17-3p) + (0.027084 × miR-3158-5p) + (0.19372 × miR-371a-5p) + (0.383034 × miR-3917) + (0.111428 × miR-3928-3p) + (-0.23688 × miR-3940-5p) + (0.217287 × miR-4449) + (0.188668 × miR-4708-3p) + (-1.673817 × miR-4739) + (-0.317227 × miR-486-5p) + (0.596774 × miR-498) + (0.854739 × miR-6088) + (-0.007404 × miR-671-5p) + (0.110257 × miR-6872-3p ) + 20.442601 |
| 1-9 | (-0.022898 × miR-1181) + (-1.163292 × miR-1228-5p) + (-0.33282 × miR-1273g-3p) + (-0.088704 × miR-17-3p) + (0.073399 × miR-3158-5p) + (0.245542 × miR-371a-5p) + (0.397722 × miR-3917) + (0.176185 × miR-3928-3p) + (-0.259566 × miR-3940-5p) + (0.247176 × miR-4449) + (0.007695 × miR-4675) + (0.211683 × miR-4708-3p) + (-1.931075 × miR-4739) + (-0.363093 × miR-486-5p) + (0.61721 × miR-498) + (0.948609 × miR-6088) + (-0.027716 × miR-671-5p) + (0.103336 × miR-6872-3p ) + 22.921317 |
|  |  |
| 6-1 (**Model TFIp ≥ 6 months**) | (0.04331 × miR-4708-3p ) + 1.0617 |
|  |  |
| 12-1 (**Model TFIp ≥ 12 months**) | (-0.0426 × miR-3141) + (0.01861 × miR-3928-3p) + (0.0129 × miR-6766-5p) + (0.0094 × miR-7108-3p ) + 0.31159 |
| 12-2 | (0.005506 × miR-1254) + (-0.233162 × miR-3141) + (0.108505 × miR-3928-3p) + (0.052679 × miR-6766-5p) + (0.046921 × miR-7108-3p ) + 0.771309 |
| 12-3 | (0.024523 × miR-1254) + (-0.370958 × miR-3141) + (0.158208 × miR-3928-3p) + (0.074012 × miR-6766-5p) + (-0.008133 × miR-6869-5p) + (0.073977 × miR-7108-3p ) + 1.267906 |
| 12-4 | (0.030583 × miR-1254) + (-0.414682 × miR-3141) + (0.174476 × miR-3928-3p) + (-0.002631 × miR-6722-3p) + (0.080808 × miR-6766-5p) + (-0.026614 × miR-6869-5p) + (0.085647 × miR-7108-3p ) + 1.635997 |
| 12-5 | (0.041917 × miR-1254) + (-0.492179 × miR-3141) + (0.201249 × miR-3928-3p) + (0.003775 × miR-4322) + (-0.052872 × miR-6722-3p) + (0.098461 × miR-6766-5p) + (-0.05535 × miR-6869-5p) + (0.105975 × miR-7108-3p ) + 2.590153 |
|  |  |
| 36-1 | (-0.0085052×miR-1254) + (-0.2186654×miR-187-5p) + (-0.0117764×miR-1908-3p) + (0.0192129×miR-191-5p) + (-0.0818024×miR-3141) + (-0.4142278×miR-3197) + (0.1690961×miR-320a) + (0.0526361×miR-342-5p) + (0.0145941×miR-4429) + (0.3025396×miR-4447) + (-0.3635017×miR-4463) + (0.0458145×miR-4515) + (0.0542341×miR-4675) + (0.0004475×miR-4732-5p) + (0.0496912×miR-486-5p) + (0.2732072×miR-557) + (-0.0545019×miR-642a-3p) + (0.0599133×miR-6766-3p) + (-0.1072596×miR-6799-5p) + (-0.0986669×miR-6808-5p) + (-0.4353737×miR-6858-5p) + (-0.0400214×miR-718) + (-0.3317855×miR-8089) + (-0.0853479×miR-887-3p ) + 11.2233496 |
| 36-2 | (-0.0100718×miR-1254) + (-0.2774778×miR-187-5p) + (0.0235554×miR-191-5p) + (-0.0858186×miR-3141) + (-0.4645236×miR-3197) + (0.1909219×miR-320a) + (0.0730775×miR-342-5p) + (0.038733×miR-4429) + (0.3572965×miR-4447) + (0.0018236×miR-4449) + (-0.566197×miR-4463) + (0.0426433×miR-4515) + (0.139225×miR-4675) + (0.0015923×miR-4732-5p) + (0.060029×miR-486-5p) + (0.3794857×miR-557) + (0.0006165×miR-6088) + (-0.0948298×miR-642a-3p) + (0.0941779×miR-6766-3p) + (-0.1842586×miR-6799-5p) + (-0.1423826×miR-6808-5p) + (-0.4693842×miR-6858-5p) + (-0.1065337×miR-718) + (-0.4048481×miR-8089) + (-0.0975639×miR-887-3p ) + 14.2793853 |
| 36-3 | (-0.010512×miR-1254) + (-0.296032×miR-187-5p) + (0.025459×miR-191-5p) + (-0.085324×miR-3141) + (-0.480216×miR-3197) + (0.197352×miR-320a) + (0.07997×miR-342-5p) + (0.008677×miR-3928-3p) + (0.045585×miR-4429) + (0.373793×miR-4447) + (0.005308×miR-4449) + (-0.629153×miR-4463) + (0.040963×miR-4515) + (0.001092×miR-4640-5p) + (0.161587×miR-4675) + (0.001617×miR-4732-5p) + (0.06312×miR-486-5p) + (0.412694×miR-557) + (0.010712×miR-6088) + (-0.108938×miR-642a-3p) + (0.102524×miR-6766-3p) + (-0.211281×miR-6799-5p) + (-0.161989×miR-6808-5p) + (0.003912×miR-6842-5p) + (-0.48001×miR-6858-5p) + (-0.128392×miR-718) + (-0.424643×miR-8089) + (-0.101338×miR-887-3p ) + 15.160137 |
| 36-4 | (-0.0108976×miR-1254) + (-0.313461×miR-187-5p) + (0.0274867×miR-191-5p) + (-0.0877817×miR-3141) + (-0.4953478×miR-3197) + (0.2058604×miR-320a) + (0.0849152×miR-342-5p) + (0.0191089×miR-3928-3p) + (0.0006009×miR-4419b) + (0.0505798×miR-4429) + (0.3888059×miR-4447) + (0.008254×miR-4449) + (-0.702695×miR-4463) + (0.0379672×miR-4515) + (0.0200086×miR-4640-5p) + (0.1783874×miR-4675) + (0.0007739×miR-4732-5p) + (0.0678916×miR-486-5p) + (0.4450908×miR-557) + (0.0250757×miR-6088) + (-0.1206386×miR-642a-3p) + (0.1110271×miR-6766-3p) + (-0.2438217×miR-6799-5p) + (-0.1850382×miR-6808-5p) + (0.0141535×miR-6842-5p) + (-0.4881161×miR-6858-5p) + (-0.1534442×miR-718) + (-0.4433555×miR-8089) + (-0.1063541×miR-887-3p ) + 16.0608247 |
| 36-5 | (-0.011304×miR-1254) + (-0.330556×miR-187-5p) + (0.029674×miR-191-5p) + (-0.090643×miR-3141) + (-0.510819×miR-3197) + (0.21427×miR-320a) + (0.0897×miR-342-5p) + (0.029023×miR-3928-3p) + (0.003306×miR-4419b) + (0.053975×miR-4429) + (0.403336×miR-4447) + (0.011158×miR-4449) + (-0.774378×miR-4463) + (0.034354×miR-4515) + (0.037603×miR-4640-5p) + (0.195169×miR-4675) + (0.072194×miR-486-5p) + (0.4778×miR-557) + (0.039272×miR-6088) + (-0.132742×miR-642a-3p) + (0.118471×miR-6766-3p) + (-0.279327×miR-6799-5p) + (-0.208812×miR-6808-5p) + (0.023642×miR-6842-5p) + (-0.497048×miR-6858-5p) + (0.001925×miR-6877-5p) + (-0.17823×miR-718) + (-0.461239×miR-8089) + (-0.111737×miR-887-3p) + (0.004446×miR-939-5p ) + 16.976658 |
| 36-6 | (-0.011751×miR-1254) + (-0.347334×miR-187-5p) + (0.032074×miR-191-5p) + (-0.0963×miR-3141) + (-0.527889×miR-3197) + (0.223442×miR-320a) + (0.094826×miR-342-5p) + (0.036391×miR-3928-3p) + (0.005184×miR-4419b) + (0.053273×miR-4429) + (0.417271×miR-4447) + (0.015554×miR-4449) + (-0.843683×miR-4463) + (-0.00283×miR-4484) + (0.029242×miR-4515) + (0.053746×miR-4640-5p) + (0.212297×miR-4675) + (0.076018×miR-486-5p) + (0.511782×miR-557) + (0.053403×miR-6088) + (-0.144789×miR-642a-3p) + (0.123975×miR-6766-3p) + (-0.319733×miR-6799-5p) + (-0.231024×miR-6808-5p) + (0.032697×miR-6842-5p) + (-0.505911×miR-6858-5p) + (0.001763×miR-6877-5p) + (0.007005×miR-7108-3p) + (-0.202289×miR-718) + (-0.479487×miR-8089) + (-0.117001×miR-887-3p) + (0.022773×miR-939-5p ) + 17.907449 |
| 36-7 (**Model TFIp ≥ 36 months**) | (-0.0125433×miR-1181) + (0.1067349×miR-1254) + (0.0015947×miR-1268b) + (-0.3771727×miR-187-5p) + (0.0360527×miR-191-5p) + (-0.0984009×miR-3141) + (0.0233745×miR-3195) + (-0.5606837×miR-3197) + (0.2415774×miR-320a) + (0.1046963×miR-342-5p) + (0.0502296×miR-3928-3p) + (0.0009486×miR-422a) + (0.0050783×miR-4419b) + (0.05179×miR-4429) + (0.4470169×miR-4447) + (0.0210486×miR-4449) + (-0.9951068×miR-4463) + (-0.0335096×miR-4484) + (0.0152364×miR-4515) + (0.0870314×miR-4640-5p) + (0.2338915×miR-4675) + (0.0864322×miR-486-5p) + (0.5827542×miR-557) + (0.0782236×miR-6088) + (-0.1712249×miR-642a-3p) + (0.1380852×miR-6766-3p) + (-0.3991616×miR-6799-5p) + (-0.2738122×miR-6808-5p) + (0.0531576×miR-6842-5p) + (-0.5248665×miR-6858-5p) + (0.033589×miR-7108-3p) + (-0.2512049×miR-718) + (-0.5161003×miR-8089) + (-0.1219326×miR-887-3p) + (0.0626341×miR-939-5p ) + 19.9189978 |
