# Supplementary material for: Circulating serum miRNAs predict response to platinum chemotherapy in high‐grade serous ovarian cancer
Source: Cancer Med. 2024 Nov 25;13(22):e70251. doi: 10.1002/cam4.70251 (PMC11588858; doi:10.1002/cam4.70251)
Supplement: Supplementary file 5 — Table S2. [file CAM4-13-e70251-s005.docx]

Supplementary Table 2.

|  | Predict TFIp | | | |
| --- | --- | --- | --- | --- |
| Model No. | > 1 month | ≥ 6 months | ≥ 12 months | ≥ 36 months |
| Model TFIp > 1 month | **0.944** | **0.663** | **0.603** | **0.618** |
| Model TFIp ≥ 6 months | **0.631** | **0.637** | **0.570** | **0.563** |
| Model TFIp ≥ 12 months | **0.757** | **0.698** | **0.705** | **0.659** |
| Model TFIp ≥ 36 months | **0.624** | **0.641** | **0.645** | **0.938** |

AUC for all subgroups based on each model.

Note: Data are shown as AUC unless otherwise noted.
